# Evolution Characteristics of Void in the Caving Zone Using Fiber Optic Sensing

**DOI:** 10.3390/s24020478

**Published:** 2024-01-12

**Authors:** Jing Chai, Fengqi Qiu, Lei Zhu, Dingding Zhang

**Affiliations:** 1 College of Energy Engineering, Xi’an University of Science and Technology, Xi’an 710054, China; chaij@xust.edu.cn (J.C.); zhangdd@xust.edu.cn (D.Z.); 2 China Coal Energy Research Institute Co., Ltd., Xi’an 710054, China; zhulei202203@126.com

**Keywords:** fiber optic sensing, overhanging rock mass collapse, void evolution, mining process, strain monitoring

## Abstract

Addressing the issue of low filling efficiency in gangue slurry backfilling due to unclear evolution characteristics of voids in the overlying collapsed rock mass during mining, this study utilizes fiber optic sensing technology to monitor real-time strain changes within the rock mass. It proposes a void zoning method based on fiber optic sensing for mining the overlying rock and, in combination with physical model experiments, systematically investigates the dimensions, distribution, and deformation characteristics of rock mass voids. By analyzing fiber optic sensing data, the correlation between the rate of void expansion and the stress state of the rock mass is revealed. The research results demonstrate that as mining progresses, the internal voids of the rock mass gradually expand, exhibiting complex spatial distribution patterns. During the mining process, the expansion of voids within the overlying collapsed rock mass is closely related to the stress state of the rock mass. The rate of void expansion is influenced by changes in stress, making stress regulation a key factor in preventing void expansion and rock mass instability. The application of fiber optic sensing technology allows for more accurate monitoring of changes in rock mass voids, enabling precise zoning of voids in the overlying collapsed rock mass during mining. This zoning method has been validated against traditional theoretical calculations and experimental results. This research expands our understanding of the evolution characteristics of voids in overlying collapsed rock mass and provides valuable reference for backfilling engineering practices and backfilling parameter optimization.

## 1. Introduction

**Figure 1:**
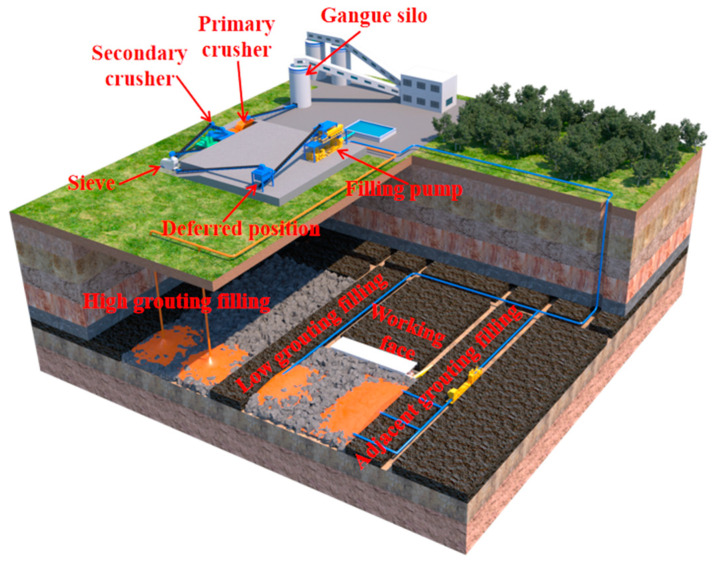
Schematic Diagram of Slurry Filling Mining.

The western region of China, as the primary coal-producing area, plays a crucial role in ensuring the nation’s independent energy supply. However, the large-scale and intensive extraction of coal resources in the western region has had a significant impact on the ecological environment. One of the major sources of ecological disruption in mining areas is coal gangue, which not only occupies a substantial amount of land but also leads to severe pollution of the air and surface water. As environmental awareness continues to grow in society, the negative effects of coal gangue emissions and resulting environmental pollution have become increasingly unacceptable. Thus, it has become imperative to find sustainable and environmentally friendly solutions for dealing with coal gangue [1,2]. In response to this challenge, Zhu Lei [3,4] and others have pioneered the concept of a slurry filling technology system, which crushes and grinds the coal gangue solid waste into tiny particles and then migrates the solid waste particles to the residual space after the collapse of underground mining with water as the carrier, pipeline as the channel, and pump as the driving force, so as to achieve the purpose of efficiently dealing with the gangue, which consists of a crushing slurry system, pipeline conveying system, and multi-bit filling system, and the principle of the coal gangue slurry filling technology. The principle of coal gangue slurry filling technology is schematically shown in Figure 1. This system utilizes the voids formed by the collapse of overlying rocks in the coal mining process for gangue disposal. The size of these voids, which serve as the space for slurry filling, directly determines the effectiveness and efficiency of the slurry filling process. These voids exhibit different distribution characteristics in different positions within the goaf. The areas near the coal mining face experience lower stress, resulting in loose rock distribution and larger inter-rock voids, making them ideal for effective slurry filling. However, as the distance from the goaf and mining face increases, the load-bearing capacity of the collapsed rock mass gradually grows, leading to the compaction of loosely piled rocks and a reduction in inter-rock voids, thereby diminishing the effectiveness of slurry filling [5,6,7]. Therefore, understanding the evolving characteristics of voids in the collapsed rock mass behind the coal mining face is of paramount importance for successful slurry filling.

Due to the “inaccessibility” of underground goaf spaces, monitoring the evolving characteristics of voids in the collapsed rock mass is challenging on-site. Currently, the primary research methods involve theoretical analysis [8], numerical simulations [9], and physical similarity modeling [10]. Among these, physical similarity modeling experiments, which create a real physical model that adheres to the principles of basic similarity, provide a more accurate representation of the engineering relationships. They have gained widespread use in the field of mining and other engineering domains. Presently, physical similarity model tests typically employ instruments such as inclinometers, total stations, pressure sensors, pressure cells, and strain gauges to monitor the deformation and stress of the physical model. However, most of these instruments provide point-based measurements, and their testing precision and sensitivity are relatively low, especially when it comes to electronic sensors, which are prone to moisture-related failures, rendering them unable to provide valid data.

Distributed fiber optic sensing technology, as an advanced monitoring technique, possesses several advantages, including corrosion resistance, compact size, lightweight, high sensitivity, high precision, real-time data acquisition, and a wide measurement range. It has been widely applied in various fields, including railways, bridges, tunnels, oil and gas pipelines, and geotechnical structures. In recent years, many experts and scholars have extended the use of this technology to the field of mining, providing essential support for safe and efficient mining operations. In on-site monitoring, Lei, W. et al. [11] investigates the pressure relief impact of mining the upper protective coal seam through the application of distributed optical fiber sensing monitoring. Additionally, Hu, T. et al. [12] involve field monitoring of roof strata movement in coal mining, utilizing distributed fiber optic sensing (DFOS) technology. Liu, J. et al. [13] explores the transformation of telecommunication fiber optic cables into distributed acoustic sensors to facilitate vibration-based monitoring of bridge health. In terms of monitoring physical similarity models, J. Chai et al. [14,15] have applied fiber optic sensing technology to investigate the deformation and stress characteristics of overlying rocks during mining, temperature/humidity field measurements in physical similarity models, and goaf pressure, achieving a series of significant results. In summary, distributed fiber optic sensing technology has made significant breakthroughs in both practical engineering applications and theoretical research.

This article focuses on a physical similarity modeling experiment conducted at the 61,601 comprehensive mining face of Longwanggou coal mine, considering the geological conditions as the engineering background. By installing vertical fiber optics inside the model, the deformation of the overlying rocks under different excavation states was monitored. The research aimed to analyze the height of rock collapses after coal mining and the evolving characteristics of voids in the rock mass behind the mining face. The findings of this study provide valuable guidance for the further application of distributed fiber optic sensing technology in on-site monitoring of voids in the collapsed rock mass resulting from coal mining.

## 2. Principle of Zoning Monitoring of Voids in Mining Overburden Rock Mass Caving Based on Fiber Optic Monitoring

### 2.1. Analysis of the Characteristics of Void Development in the Overlying Rock Collapse Zone along the Mining Face

**Figure 2:**
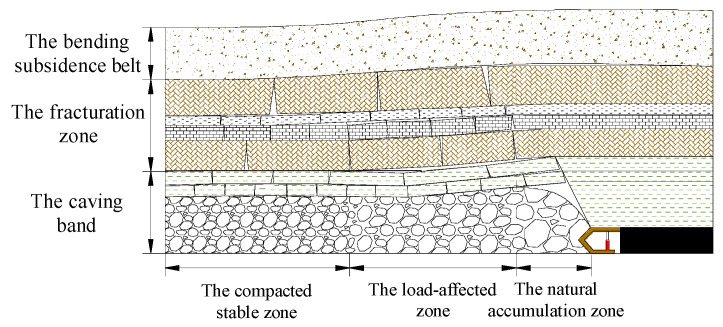
Partition Characteristics of Void Development in the Overlying Rock Collapse Zone.

During the advancement of the mining face, the key stratum, as the load-bearing structure of the overlying rock layers, undergoes a dynamic evolution process of stability, rupture, hinge, and re-stabilization under the influence of its own weight and the load from the overlying rock layers. With the repeated occurrence of this process, the voids in the collapsed rock mass beneath the key stratum along the mining face exhibit distinct partition characteristics, namely: the natural accumulation zone, the load-affected zone, and the compacted stable zone [16,17,18], as illustrated in Figure 2.

(1) Characteristics of the Natural Accumulation Zone

Within a certain range behind the advancing mining face, the overlying key stratum is in a cantilevered state, and there is a gap between the collapsed rock mass and the key stratum above it. It is not affected by the stress from the basic roof and its overlying rock load but only bears the stress caused by the self-weight of the collapsed rock layer. Therefore, the rock mass in this area is in a natural accumulation state. The degree of rock fragmentation is high in the natural accumulation zone, and there are significant gaps between the broken rock fragments, providing ample space for slurry filling. This zone is the primary target area for slurry filling and moves forward periodically with the advancement of the mining face.

The length of the natural accumulation zone in the goaf can be calculated using the ultimate shear block length during the cantilevered beam failure of the key roof:

(1) $$L_{a}=\frac{2\tau_{\max}}{3(1-\xi \tan\varphi )\left(\gamma_{j}+\frac{\gamma_{s}}{J_{z}}\right)}$$

where *L* is the length of the natural accumulation zone, m; *τ*_max is the maximum shear stress of the rock layer, MPa; *φ* is the average internal friction angle of the rock layer, (°); *γ_s_* is the bulk density of the sand layer, KN·m^−3^; *γ_r_* is the bulk density of the rock layer, KN·m^−3^.

(2) Characteristics of the Load-Affected Zone

In the transitional area far from the mining face coal wall towards the central part of the goaf, the key stratum above the collapsed rock mass undergoes rupture, forming a “block beam” structure [19,20]. With the continuous advancement of the mining face, it exerts a certain rotational angle on the collapsed rock mass until the entire broken rock block acts on the collapsed rock mass and no longer sinks. In this area, the collapsed rock mass is subjected to the stress from the basic roof and its overlying rock load, resulting in reduced voids compared to the natural accumulation zone. However, since it is not fully compacted, slurry can still be filled in this area, making it suitable for slurry filling. The load-affected zone also moves forward periodically with the advancement of the mining face, and the voids in the collapsed rock mass gradually become compressed. When the length of the load-affected zone reaches its maximum, the voids in the goaf will be fully compacted.

The length of the load-affected zone in the goaf can be calculated using the surrounding rock load model:

(2) $$L_{b}=\left\{\begin{array}{l} H\tan\beta -L_{a}\;\;\frac{H}{l}\leq \frac{1}{2\tan\beta }\\ l-\frac{l}{4H\cdot \tan\beta }-L_{a}\;\;\frac{H}{l}>\frac{1}{2\tan\beta } \end{array}\right.$$

where *L_b_* is the length of the load-affected zone, m; *H* is the depth of the working face, m; *β* is the coal-rock support extension angle, (°).

(3) Characteristics of the Compacted Stable Zone

Beyond the load-affected zone, the key stratum above the collapsed rock mass has undergone stable movement, and the stress has largely returned to the original rock stress. The rock mass in this area has been mostly compacted, making it unsuitable for effective slurry filling. The extent of the compacted stable zone increases along the advancement of the mining face.

The length of the compacted stable zone in the goaf can be calculated as

(3) $$L_{c}=\frac{L}{2}-L_{a}-L_{b}$$

where *L_c_* is the length of the compacted stable zone, m; *L* is the length of the mining face advancement, m.

In summary, the movement state of the key stratum overlying the goaf plays a controlling role in the development of void size in the collapsed rock mass. If the movement state of the key stratum can be monitored during coal seam extraction, it can indirectly reflect the size of voids in the goaf’s collapsed rock mass. Fiber optic sensing technology, known for its high sensitivity and precision, has been widely applied to monitor overlying rock deformation during mining, yielding a series of achievements. Therefore, distributed fiber optics can be used to monitor the movement process of the overlying rock.

### 2.2. Principle of Fiber Optic Monitoring for Gap Zoning in Mining Overburden Collapse Rock Mass

To analyze the force characteristics of vertical fiber optics during overlying rock movement in the coal seam extraction process, the following assumptions are made: there is direct and full contact between the rock layer and the optical fiber with no adhesive material or gaps. The optical fiber is well-coupled with the embedded rock layer. The optical fiber is considered a single unit, with the core and cladding having the same mechanical properties.

Under these assumptions, the forces on the optical fiber embedded in the rock layer can be divided into shear stress along the axis of the fiber and compressive stress along the radial direction of the fiber. When the rock layer has not deformed, the optical fiber experiences compressive stress (*σ_r_*) from the rock layer, tensile pre-stress (*σ_a_*) applied uniformly on the optical fiber before embedding, and shear stress (*τ_f_*) on the optical fiber due to friction between the rock layer and the optical fiber, which results from the optical fiber’s rebound effect as it is embedded in the rock layer after tension. For a fiber element of length *dx*, the equilibrium equation of forces along the axial direction of the optical fiber can be written as

(4) $$\pi r^{2}\sigma_{a}(x)=\pi r^{2}\left[\sigma_{a}(x)+d\sigma_{a}(x)\right]+2\pi r(x,r)d(x)$$

where *r* is the radius of the optical fiber; *τ* is the shear stress on the optical fiber; *σ_a_* is the axial stress on the optical fiber.

**Figure 3:**
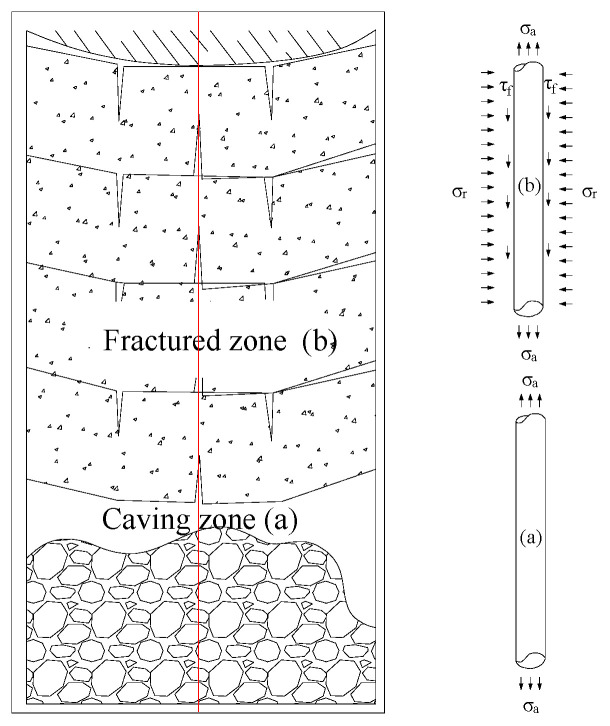
Characteristics of Forces on Vertical Fiber Optics.

After coal seam extraction, as the overlying rock continuously collapses from the bottom to the top, the rock layers exhibit distinct zonal collapse features with corresponding zonal stress changes within the rock layers due to mining. In this context, the mechanical force state of the rock layers and the optical fiber within the collapse and fracture zones is discussed. A mechanical analysis model for the deformation of rock layers in the collapse and fracture zones with vertical fiber optics is established, as shown in Figure 3.

When the optical fiber is in the fracture zone, the lower rock layer collapses, causing the upper rock layer to rupture and move downward under the influence of gravity. The horizontal radial stress σr on the optical fiber increases, primarily due to the compressive force exerted by the moving lower rock layer on the optical fiber. This results in a decrease in the axial shear stress τf on the optical fiber, with part of the decrease attributed to the frictional force generated by the rock layer’s compressive force, which impedes the optical fiber from moving downward under the influence of gravity. At this point, there is still the pre-existing axial stress σa on the optical fiber, but its magnitude is much smaller than the stress induced by rock layer deformation.

Based on the above analysis, it is evident that the movement of the overlying strata’s key layers plays a controlling role in the development of voids in the goaf area. If the movement status of the overlying strata’s key layers can be monitored during coal seam extraction, it can indirectly reflect the size of voids in the collapsed rock mass within the goaf. Optical fibers, as highly sensitive and precise sensors, have been widely utilized in monitoring the deformation of overlying strata during mining operations, leading to a series of achievements. Therefore, the size of the voids in the collapsed rock mass within the goaf can be characterized by using distributed optical fiber monitoring of the movement of overlying strata during mining. When the optical fiber is in the natural accumulation zone, the key layers behind the working face have not completely fractured. As the working face continues to advance, the tensile stress generated by the “cantilever beam” structure formed by the key layers rapidly increases, causing a rapid rise in strain values in the optical fiber. When the optical fiber is in the load-affected zone, the “cantilever beam” structure formed by the key layers has completely fractured, and with the continuous excavation of the working face, under the influence of the superimposed load, the fractured key layers begin to experience a reduction in the tensile stress exerted on the optical fiber, leading to a decrease in strain values. When the optical fiber is in the recompacted zone, the fractured key layers have stabilized, no longer imposing tensile stress on the optical fiber, and the strain values in the optical fiber also begin to stabilize.

## 3. Similar Model Test for Overlying Rock Movement

### 3.1. Overview of Similar Simulation Test

The Longwanggou Minefield is located 120 km east of Ordos City in Inner Mongolia Autonomous Region, in the northern part of the Zheng’er Coalfield, with a field area of 51.149 km^2^ and a designed annual production capacity of 10 million tons. The main coal seam for mining is Coal Seam No. 6, and the mine adopts the comprehensive shearer top coal caving mining method, managing the overlying strata through full collapse. In this study, the geological conditions of the 61,601 working face at Longwanggou were selected as the engineering background for similar simulation tests. The 61,601 working face has a strike length of 615 m, a dip length of 254.6 m, an average coal thickness of 23.15 m, and an average coal seam dip of 2.75°, which is nearly horizontal.

Based on the geological column diagram of the overlying strata of Coal Seam No. 6 and the physical–mechanical parameters of the rock layers, the test used river sand, gypsum, and talcum powder as similar materials. These materials were mixed with water in accordance with certain similarity ratios and then layered into a two-dimensional model frame for compaction. Mica powder was used to create layering between the different rock layers. The structure of the model rock layers and the composition of the similar materials are shown in Table 1, and the basic parameters of the model are presented in Table 2.

### 3.2. Test Measurement Systems

#### 3.2.1. Overlying Rock Settlement Displacement Monitoring System

**Figure 4:**
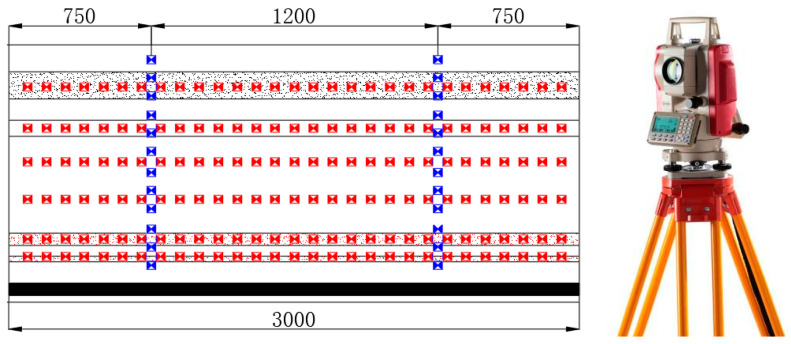
Total station survey point layout.

To study the damage characteristics and movement deformation patterns of the overlying rock during the mining process, a Leica TS02 optical total station was used for measurements. Six horizontal measuring lines and a total of 194 total station displacement measurement points were set up on the model surface. The distance of 100 mm was maintained between two adjacent points. The arrangement of the measurement lines and points concerning the distance from the coal seam roof and the left side of the model frame is shown in Figure 4. The measuring lines were denoted as A, B, C, and so on, and the points were numbered from 1 to 25 from top to bottom, resulting in designations such as A1, A2, A3…A25. The initial coordinates of each measurement point were recorded before the model excavation. As the mining face advanced, the overlying rock layers would either collapse or move. In areas with apparent deformation of the overlying rock, coordinates of the measurement points were recorded using the total station.

#### 3.2.2. Distributed Fiber Optic Testing System

(1) Testing System

**Figure 5:**
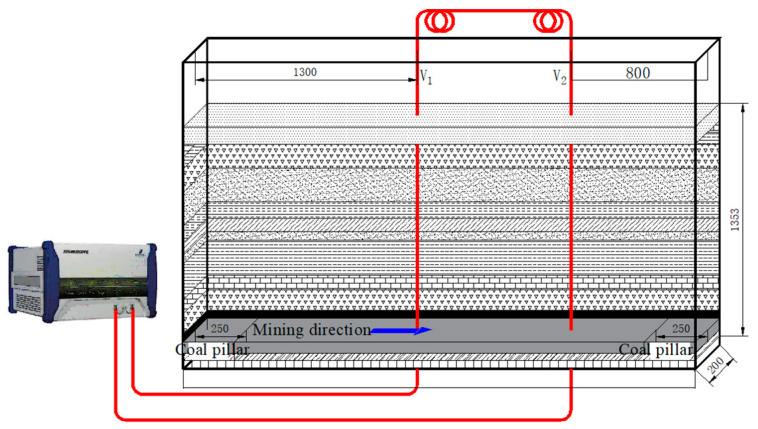
Similar model fiber optic test system.

In this experiment, embedded optical fibers were used to monitor the movement of the overlying rock. As shown in Figure 5, before laying the similar materials, the optical fibers were secured within the rock layers, and a certain pre-stress was applied to ensure that the optical fibers were taut. The parameters of the NBX-6055 fiber optic analyzer were configured for the accurate measurement of internal deformation in the rock layers. Two vertical fibers, V1 and V2, were located at positions 1300 mm and 800 mm from the left and right sides of the model, respectively. A pre-stress was applied to the fibers to avoid difficulties in positioning caused by bending during the compaction of similar materials, and to ensure good coupling with the model for coordinated deformation. The main technical performance parameters of the NBX-6055 fiber optic analyzer are detailed in Table 3.

(2) Uncertainty Analysis

**Figure 6:**
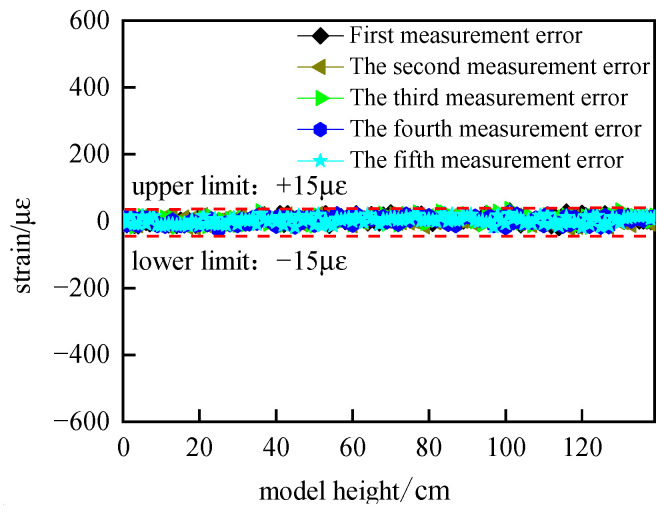
Distributed fiber optic strain uncertainty test.

Five repetitive measurements of the optical fiber embedded in the model were performed to verify the uncertainty of the optical fiber test system before model mining. The strain uncertainty due to the system error is obtained from the difference between the last four measurements and the first one, and the test results are shown in Figure 6, where the horizontal coordinate is the length of the model and the vertical coordinate is the strain on the fiber.

As can be seen from Figure 6, the five test results are basically the same, and the measured strain fluctuates within the range of 15 με. Within the test range, the uncertainty of the test system caused by 15 με has a very small effect on the experimental results, which is basically negligible, and therefore, the test system is reliable.

#### 3.2.3. Bottom Plate Pressure Testing System

**Figure 7:**
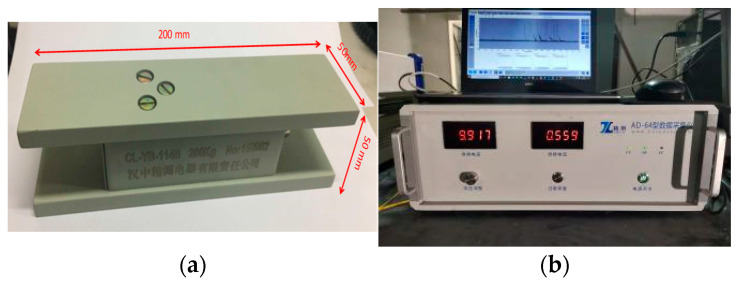
Pressure testing system. (**a**) Pressure Sensor. (**b**) Pressure testing device.

The experiment used a CL-YB-114 pressure sensor to measure changes in the load-bearing pressure of the model. Before laying the similar materials, pressure sensors were placed at the bottom of the model frame. A total of 60 pressure sensors were placed on the mine’s working face floor, numbered from 0 to 60 from the left end to the right end of the model. The pressure changes on the model’s floor were recorded using a multi-channel pressure data acquisition system as the mining face advanced, after each excavation, to observe the pressure exerted by the overlying strata. The pressure sensors and data acquisition system are depicted in Figure 7.

### 3.3. Test Procedure and Observations

The model was constructed with protective coal pillars of 30 cm on each side, and it was excavated for a total length of 240 cm, with excavation steps of 4 cm, resulting in 60 steps in total. During the experiment, data were collected and recorded at each step, both before and after excavation, using the distributed fiber optic demodulator and the bottom plate pressure data acquisition system.

**Figure 8:**
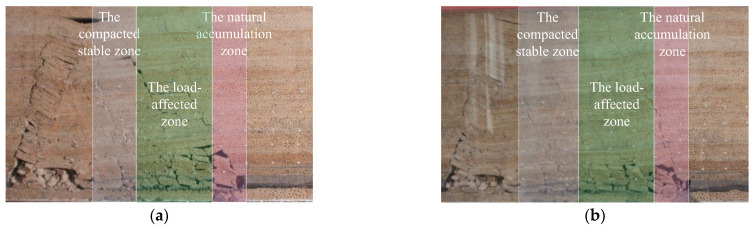
Model Excavation Process. (**a**) The 37th Excavation. (**b**) The 44th Excavation.

The states of overlying rock collapse at the 37th and 44th excavation steps are shown in Figure 7. At the 37th step, a large area of the overlying rock layer collapsed due to the fracture of the old roof, with a collapsed height of 40 cm, a collapsed upper boundary width of 30 cm, and free spaces in the collapsed rock layers reaching a maximum height of 2.5 cm. When the working face reached the 44th step, the collapsed rock layer had a height of 65 cm, a width of 37 cm, and the free space continued to develop upwards due to rock fragmentation. The free space was reduced due to rock dilation, with a height of 1 cm. Clear zones could be observed above the collapse zone, namely, the natural accumulation zone, the load-affected zone, and the compacted stable zone, as shown in Figure 8.

## 4. Experimental Results Analysis

### 4.1. Analysis of Overlying Strata Settlement Displacement and Porosity

**Figure 9:**
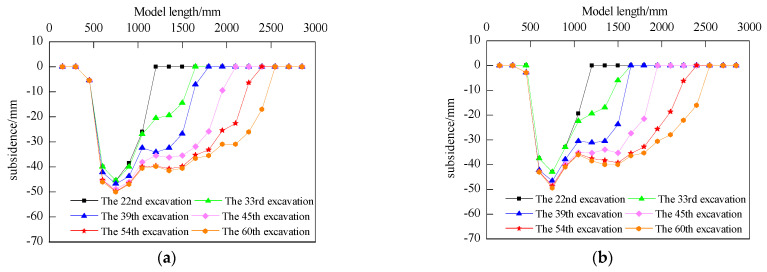
Roof Layer Subsidence Curves. (**a**) 99 mm above the coal seam. (**b**) 146 mm above the coal seam.

**Figure 10:**
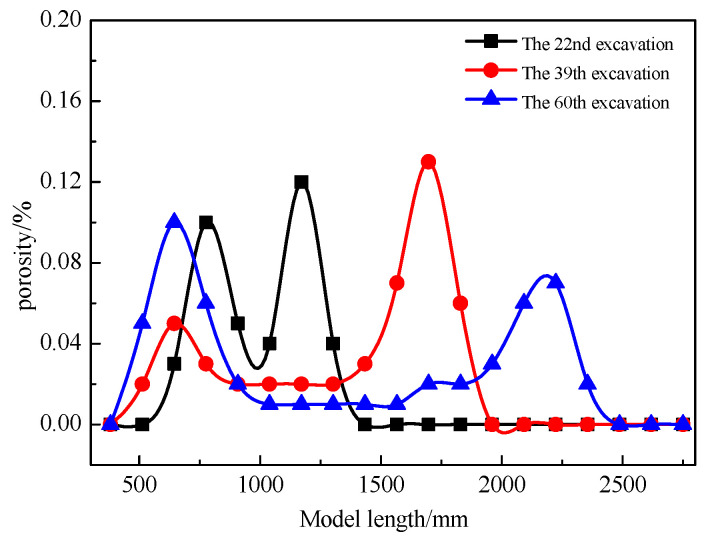
Porosity Distribution Curves in the Collapse Zone.

In order to understand the porosity distribution characteristics of the overlying strata collapse zone after coal excavation, coarse sandstone layers located 99 mm and 146 mm above the coal seam were selected for monitoring. Figure 9 shows the subsidence curves of the roof layers at different locations during the mining process. The maximum subsidence of the coarse sandstone layer located 99 mm above the coal seam is 52 mm, while the maximum subsidence of the coarse sandstone layer located 146 mm above the coal seam is 50 mm. By subtracting the distance between the two adjacent measuring points on the 99 mm and 146 mm measuring lines above the coal seam after mining from the distance before mining, and comparing the difference to the distance before mining, the distribution curve of void ratio in the overlying rock collapse zone under different excavation times can be obtained, as shown in Figure 10.

From the porosity distribution curves in the collapse zone, it can be observed that the change trend in porosity along the direction is consistent for different excavation cycles, showing an overall “saddle-shaped” distribution. This means that the porosity is higher near the coal pillars on both sides of the goaf, decreases in the middle area, and peaks near the working face. As the working face advances, the peak in porosity gradually moves forward, and the porosity behind the peak decreases. Based on the distance from the working face, these zones can be classified as the natural accumulation zone (0–100 mm behind the working face), the load-affected zone (100–400 mm behind the working face), and the compacted stable zone (beyond 400 mm behind the working face).

### 4.2. Characteristics of Overlying Rock Deformation Zoning

**Figure 11:**
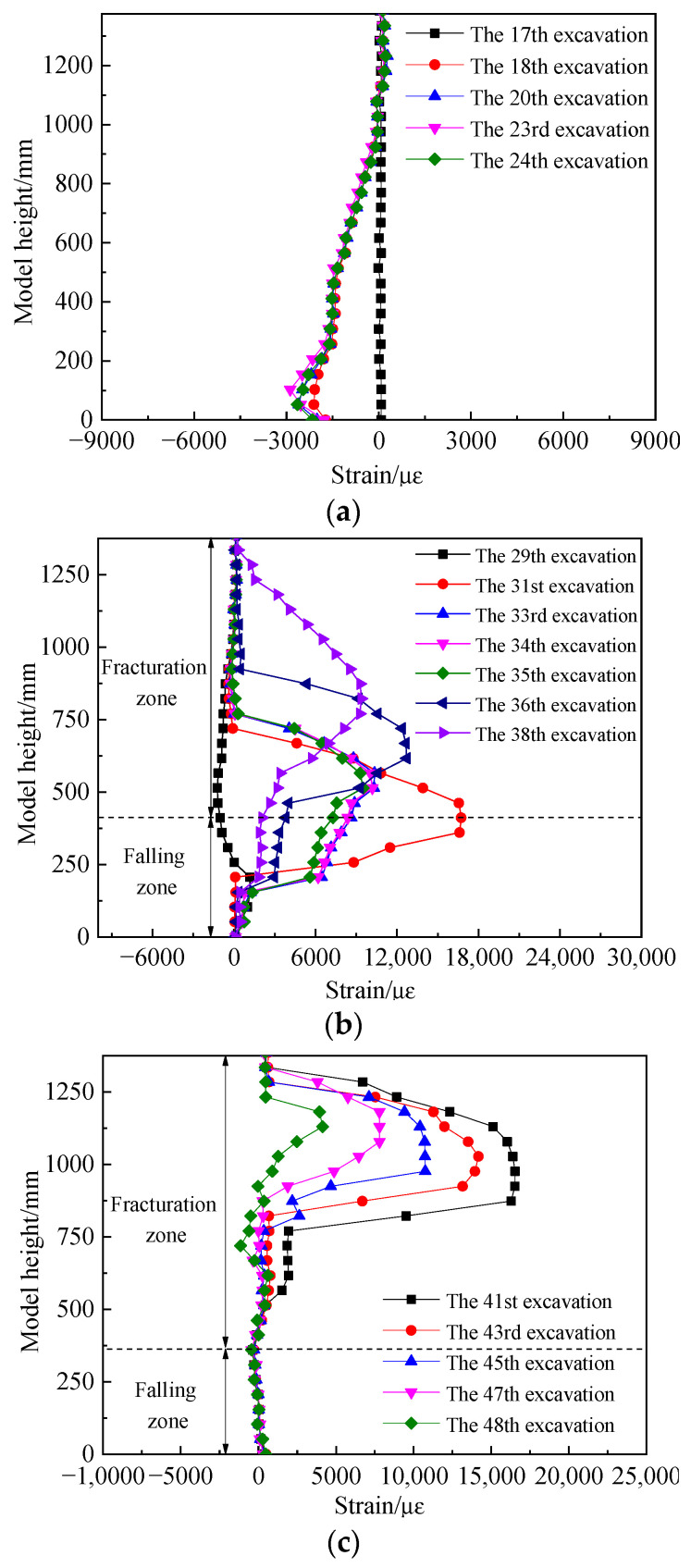
Vertical Fiber V1 Test Results. (**a**) Working face near the fiber optic cable. (**b**) Working face over the fiber optic cable. (**c**) Working face away from the fiber optic cable.

To understand the variation in fiber optic strain values as the working face approaches, passes through, and moves away from the fiber optic cable throughout the entire process, we focus on the results from the V1 fiber optic cable. Figure 11 shows the strain monitoring results for V1 during the mining process. The vertical axis represents the model’s height, and the horizontal axis represents the strain monitoring values provided by the fiber optic cable. The positive strain values indicate fiber tension, while the negative values indicate fiber compression.

When the working face is close to the V1 fiber optic cable, as shown in Figure 11a, there is little change in fiber optic strain values before the 17th excavation cycle, indicating that the rock mass around the fiber is not significantly affected by the advance support pressure from the working face. The strain values become negative during the 18th excavation cycle, and the change in fiber optic strain occurs at a height of 873 mm, with a maximum strain of −2108.46 με. This indicates that the overlying rock within a height of 873 mm above the coal seam is under compression. As the excavation continues, the fiber optic strain peak gradually increases, reaching its maximum during the 23rd excavation cycle at a height of 80 mm from the V1 fiber. This trend correlates with the changes in advance support pressure from the working face.

When the working face passes over the V1 fiber optic cable, as shown in Figure 11b, the strain values increase as the face advances until they reach a maximum at 644 mm above the coal seam during the 31st excavation cycle. Subsequently, as the working face advances, the strain values start to decrease gradually. By the 36th excavation cycle, the range in strain variation extends to 924 mm in height, indicating that the fractures around the V1 fiber optic cable have developed to a height of 924 mm, and these fractures continue to propagate upward as the working face advances.

When the working face moves away from the V1 fiber optic cable, as shown in Figure 11c, the strain values within the range of 0–462 mm (collapse zone) become negative, indicating that the rocks in this zone are consolidating. Within the range of 462–1353 mm (fracture zone), the strain values are positive, indicating tension in the rocks. As the working face continues to advance, the strain values in the range of 0–462 mm stabilize, while those in the range of 462–1353 mm gradually decrease and stabilize. This suggests that the rocks around the V1 fiber optic cable have undergone full deformation, and further mining no longer significantly affects their deformation.

### 4.3. Characteristics of Stress Zoning in Collapse Zone

**Figure 12:**
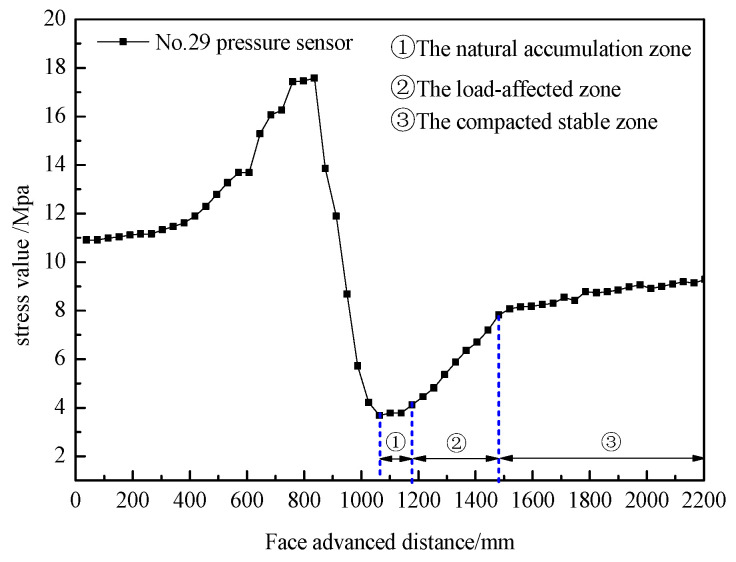
Stress Distribution Curve for the Floor Pressure Sensor.

Figure 12 shows the variation in stress as the working face advances at the location of pressure sensor No. 29. The results reveal that as the working face advances, stress changes as follows: in the range from 0 to 608 mm, stress remains almost unchanged. Between 608 and 836 mm, stress rapidly increases, reaching a peak of 17.58 MPa at 836 mm. This increase is primarily due to the influence of advance support pressure from the working face. In the range from 836 to 1064 mm, the stress curve rapidly decreases. In the range from 1064 to 1178 mm, stress remains at its lowest. This is because the working face has just passed the location of pressure sensor No. 29, and the coal seam forms a “cantilever beam” structure, resulting in a low-stress area at this location. In the range from 1178 to 1482 mm, stress gradually increases, as the “cantilever beam” structure above the coal seam has fully ruptured and is rebounding under the effect of the overlying load. Beyond 1482 mm, the stress remains relatively constant, indicating the recompaction of the collapsed rock mass.

Based on this analysis, the working face sequentially forms zones of low stress, rising stress, and stable stress behind the working face, which corresponds to the natural accumulation zone (0–114 mm behind the working face), the load-affected zone (114–418 mm behind the working face), and the compacted stable zone (beyond 418 mm behind the working face).

## 5. Characterizing Fiber Optic Data in the Collapse Zone

**Figure 13:**
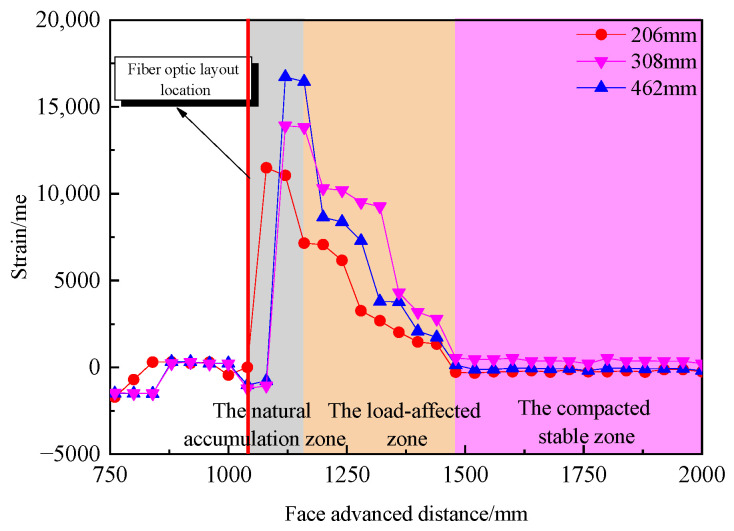
Fiber Optic Strain Distribution Curves.

In order to further analyze the relationship between the strain variation in the V1 fiber optic cable in the direction of advancement and the evolution of porosity in the collapse zone during the working face advance, the fiber optic strain data at model heights of 206 mm, 308 mm, and 462 mm were selected, and the strain variation curves are shown in Figure 12. From Figure 13, it is evident that the fiber optic strain values remain stable until the working face reaches 1160 mm. Beyond that point, the strain values increase, reaching a peak strain of 11,491 με at a height of 206 mm, 13,905 με at a height of 308 mm, and 16,725 με at a height of 462 mm. This indicates that the “cantilever beam” structure above the coal seam starts to fracture, although it is not fully ruptured at this point. As the working face advances further, the strain values decrease, suggesting complete rupture of the “cantilever beam” structure. The process of rupture is gradual due to the rock fragmentation in the collapse zone. Beyond 1480 mm, the fiber optic strain values remain constant, indicating that the rock mass in the collapse zone has been largely recompacted.

In summary, the strain variation of the V1 fiber optic cable in the advancement direction closely correlates with changes in the porosity of the collapse zone. Based on the strain distribution curves, the region with rapidly increasing strain corresponds to the natural accumulation zone (0–120 mm behind the working face), the region with gradually decreasing strain corresponds to the load-affected zone (120–420 mm behind the working face), and the region with stable strain corresponds to the compacted stable zone (beyond 420 mm behind the working face). These findings align with the zones identified based on the overlying strata subsidence and floor pressure test results.

## 6. Conclusions

(1) This study delves into the evolution characteristics of cavity collapse in overhanging rock masses during the mining process through the application of fiber optic sensing technology. Real-time monitoring using fiber optic sensing reveals the dynamic changes in internal rock cavities, the proposed calculation method for zoning overlying rock cavities based on fiber optic sensing is reliable, providing essential information for the development and assessment of coal mine slurry filling solutions.
(2) During the mining process, the expansion of cavities within overhanging rock masses is closely linked to the stress state of the rock. The rate of cavity expansion is influenced by stress variations, and stress control may become a crucial factor in preventing cavity expansion and rock mass instability.
(3) The changes in cavity zones at different stages of overhanging rock mining can be characterized through segmented strain curves obtained via fiber optic monitoring. Fiber optic strain monitoring results accurately delineate the boundaries of the natural accumulation zone, the load-affected zone, and the compacted stable zone.

## Figures and Tables

**Table 1:** Structural parameters of overburden.

Serial Number	Rock Type	Thickness /m	Cumulative Thickness /m	Bulk Densit /kN·m^−3^
18	Loess	23.00	412	17.9
17	Fine Sandstone	22.38	389	26.5
16	Coarse Sandstone	19.25	366.62	25.6
15	Fine Sandstone	42.03	345.51	26.5
14	Coarse Sandstone	35.22	303.48	25.6
13	Fine Sandstone	25.07	268.26	26.5
12	Sandy Mudstone	13.84	243.19	26.0
11	Fine Sandstone	14.03	229.35	26.5
10	Sandy Mudstone	21.88	209.46	26.0
9	Coarse Sandstone	25.51	184.62	25.6
8	Mudstone	11.43	159.11	23.3
7	Coarse Sandstone	24.71	139.41	25.6
6	Sandy Mudstone	5.36	113.4	26.0
5	Coarse Sandstone	42.43	108.04	25.6
4	Sandy Mudstone	4.85	51.5	26.0
3	Coal #6	19.60	31.6	14.0
2	Mudstone	3.14	10.3	23.3
1	Coarse Sandstone	7.16	7.16	25.6

**Table 2:** Basic parameter table for the physical similarity model.

Project	Parameter	Project	Parameter
Model Length	300 cm	Excavation Distance	240 cm
Model Thickness	20 cm	Model Boundary	30 cm
Model Height	135.3 cm	Excavation Steps	60
Coal Thickness	6.7 cm	Single Excavation Distance	4 cm
Geometric Ratio	1:300	Excavation Time Interval	0.5 h
Bulk Density Ratio	1.56:1	Excavation Time	30 h
Stress Ratio	380:1	Upper Load	0 MPa

**Table 3:** Main technical performance indicators of NBX-6055.

Parameter	Technical Specifications
Measurement Range (km)	0.05 to 25
Pulse Width (ns)	0.5, 1, 2, 5, 10
Spatial Resolution (cm)	5, 10, 20, 50, 100
Strain Measurement Accuracy (με)	±7.5
Temperature Measurement Accuracy (°C)	±0.75
Strain Monitoring Range (με)	−3000 to +4000
Temperature Monitoring Range (°C)	−270 to +800

## Data Availability

The data used to support the findings of this study are available from the corresponding author upon request.
